# Reference-free transcriptome signatures for prostate cancer prognosis

**DOI:** 10.1186/s12885-021-08021-1

**Published:** 2021-04-12

**Authors:** Ha T.N. Nguyen, Haoliang Xue, Virginie Firlej, Yann Ponty, Melina Gallopin, Daniel Gautheret

**Affiliations:** 1 Institute for Integrative Biology of the Cell, UMR 9198, CEA, CNRS, Université Paris-Saclay, Gif-Sur-Yvette, France; 2 Institute of Biology, Université Paris Est Creteil, Creteil, Creteil, France; 3 LIX CNRS UMR 7161, Ecole Polytechnique, Institut Polytechnique de Paris, Palaiseau, France

**Keywords:** Reference-free transcriptomic, Supervised learning, Prostate cancer signature

## Abstract

**Background:**

RNA-seq data are increasingly used to derive prognostic signatures for cancer outcome prediction. A limitation of current predictors is their reliance on reference gene annotations, which amounts to ignoring large numbers of non-canonical RNAs produced in disease tissues. A recently introduced kind of transcriptome classifier operates entirely in a reference-free manner, relying on k-mers extracted from patient RNA-seq data.

**Methods:**

In this paper, we set out to compare conventional and reference-free signatures in risk and relapse prediction of prostate cancer. To compare the two approaches as fairly as possible, we set up a common procedure that takes as input either a k-mer count matrix or a gene expression matrix, extracts a signature and evaluates this signature in an independent dataset.

**Results:**

We find that both gene-based and k-mer based classifiers had similarly high performances for risk prediction and a markedly lower performance for relapse prediction. Interestingly, the reference-free signatures included a set of sequences mapping to novel lncRNAs or variable regions of cancer driver genes that were not part of gene-based signatures.

**Conclusions:**

Reference-free classifiers are thus a promising strategy for the identification of novel prognostic RNA biomarkers.

**Supplementary Information:**

The online version contains supplementary material available at (10.1186/s12885-021-08021-1).

## Introduction

The outcome of human cancer can be predicted in part through gene expression profiling [1–3]. Outcome prediction is particularly important in prostate cancer (PCa), where distinguishing indolent from aggressive tumors would prevent unnecessary treatment and improve patients’ quality of life. However, currently there is no reliable signature of aggressive prostate cancer. Pathologists classify prostate tumor biopsies using scoring systems such as the Gleason score that evaluates tumor differentiation and Tumour, Node, Metastasis (TNM) staging that evaluates tumor extent and propagation. Gleason, TNM and Prostate-specific antigen (PSA) levels can be combined into a low, medium or high risk status [4]. Several studies used gene expression profiles to derive predictors of Gleason score or risk [5–8]. Other studies predicted actual clinical progression (tumor recurrence or metastasis) after several years of patient followup. Clinical progression can be evaluated either indirectly through monitoring of PSA levels (BCR=biochemical relapse) [9–12] or upon direct clinical observation [13–16]. Gene expression predictors usually take the form a of signature, that is a set of genes or transcripts and associated coefficients of a model that can be used to predict risk or outcome from a patient sample. Commercial tests such as Decipher and Oncotype DX predict prostate cancer risk based on gene expression. However these are still not recommended for routine use [17]. In general, the prostate cancer community has progressed pretty well at identifying low and high risk patients, but men with mid-range risk face more uncertainty and would most benefit from improved tests.

Gene expression profiling of prostate biopsies is performed either using DNA microarrays [13–16] or high throughput RNA sequencing (RNA-seq) [5–8]. An important advantage of RNA-seq is its ability to identify novel genes or transcripts, which can in principle be incorporated into predictive signatures. However, RNA-seq analysis is usually performed in a “reference-based” fashion, ie. by using RNA-seq reads to quantify a predetermined set of transcripts. This amounts to using RNA-seq in the same way as a microarray that only quantifies a predetermined set of probes. Yet, there is abundant evidence that non-reference RNAs are frequent in disease tissues and may constitute clinically useful biomarkers [18]. Therefore one may expect that prognostic models incorporating non-reference RNAs may carry substantial benefits.

Our group [19, 20] and others [21] introduced new k-mer based strategies to analyse RNA-seq data in a “reference-free” manner, that is without mapping sequence reads to a predefined set of genes or transcripts. K-mers are sub-sequences of fixed length which are extracted and quantified from sequence files. When applied to medical RNA-seq datasets using appropriate statistical methods, this strategy identifies any sub-sequence whose increased abundance is associated to a given clinical label. This may include novel splice variants, long non-coding RNAs (lncRNAs) or RNAs from repeated retroelements [19, 20] which are ignored by conventional protocols based on reference gene annotations.

Although attractive in principle, k-mer derived prognostic signatures pose two major challenges. First, a single RNA-seq dataset commonly contains tens to hundreds of millions distinct k-mers. Therefore false positive and replicability issues encountered with gene expression profiles [22–25] are expected to worsen with k-mer count matrices. The second challenge is related to the transfer of a k-mer signature across independent datasets. Signatures inferred from an initial discovery set are expected to generalize to any independent dataset. In the absence of a unifying gene concept, independent validation requires matching signature k-mers to read sequences from the new dataset. This may cause significant signal loss if sequencing or library preparation technologies differ.

Our main objective here was to compare the characteristics and performances of reference-based and reference-free classifiers for PCa risk and relapse prediction. We built both types of classifiers using the same discovery dataset and assessed their performances in independent datasets using equivalent pipelines and parameters. For the reference-free approach, this required special developments to reduce the number of variables and to transfer expression measures between datasets. We present below a detailed analysis of the relative performances and sequence contents of the different classifiers and discuss possible future developments to improve performances of models.

## Materials and methods

### Data acquisition and outcome labelling

We used tumor samples from the TCGA-PRAD data collection [26] (N=505) for signature discovery. The resulting classifiers were then assessed in two independent datasets, from the Canadian Prostate Cancer Genome Network (ICGC-PRAD-CA) [27] (N=148) and from the Portuguese Oncology Institute’s “Porto” cohort, analyzed in Stelloo et al. [28] (N=91). All three datasets were produced from radical prostatectomies and used similar technologies for library preparation (frozen samples, poly(A)+ RNA selection) and Illumina sequencing, however they differed by read-size, read depth, strandedness and use of single or paired ends sequencing (Table 1).

**Table 1:** Characteristics of prostate tumor RNA-seq datasets

Study	RNA-seq library type	Reads/sample	*#*Tumor samples	Risk		Relapse	
				LR	HR	NO	YES
TCGA-PRAD	Poly(A)+ unstranded 2x50nt	130M	505	134	240	56	58
ICGC-PRAD	Poly(A)+ stranded 2x100nt	313M	148	40	23	49	7
STELLOO	Poly(A)+ stranded 1x65nt	20M	91			43	48

TCGA-PRAD RNA-seq data were retrieved from dbGAP accession phs000178.v9.p8 with permission. ICGC-PRAD-CA RNA-seq data (EGAD00001004424) were downloaded from the European Genome-Phenome Archive (EGA) with permission. The RNA-seq files from the “Porto” cohort [28] were retrieved from GEO, under accession GSE120741. Clinical information was retrieved from Liu et al. [29] for TCGA-PRAD, from Fraser et al. [27] for ICGC-PRAD and from sample metadata of GEO accession GSE120741 for Stello et al. [28].

We built predictors for risk and relapse using two-class prediction models. To achieve a clear separation between the two classes, we only focused on high risk (HR) samples versus low risk (LR) samples, ignoring the medium risk, and we focused on relapse prior to a given year and non-relapse after a given year. For this reason, only a fraction of samples could be labelled for a given class in each set. Risk information was not available in the Stelloo dataset and relapse labelling on the ICGC dataset led to a small validation set (only 7 relapse samples).

We classified tumor specimens into low-risk and high-risk groups using an adaptation of d’Amico’s classification which does not take into account the PSA rate but only the anatomo-pathological data on the basis of Gleason and TNM features as performed previously [20]. Tumors with Gleason score 6/7 (3+4) and TNM stage pT1/2 were classified as low risk. Tumors with Gleason score 8/9 and/or TNM stage pT3b/4 were defined as high-risk. Tumors classified as pT3a, pT1 or (pT2 and Gleason (4+3)) were considered as intermediate and excluded from the analysis. 374 TCGA-PRAD tumors and 63 ICGC-PRAD-CA tumors could be labelled for LR or HR. We could not obtain Gleason/TNM scores for Stelloo et al, hence we did not annotate risk for this cohort.

For relapse analysis, we distinguished patients with biochemical relapse (BCR) and time to BCR <2yr and patients with no BCR after 5 years or longer, except for Stelloo et al. where only precomputed relapse data was available with cutoffs at 5yr and 10yr, respectively (Table 2). BCR information was obtained from Table S1 of Liu et al. [29] for TCGA-PRAD and from table S1 (PFS field) of Fraser et al. [27] for ICGC-PRAD. Precomputed relapse data for Stelloo et al. was taken from SRA accession PRJNA494345.

**Table 2:** Relapse group definitions

Relapse group	TCGA-PRAD	ICGC-PRAD	STELLOO
Relapse (YES)	PFS = 1 and	BCR = “Yes” and	BCR = “Yes” and
	PFS.time <2yr	BCR.time <2yr	BCR.time <5yr
Non relapse (NO)	PFS = 0 and	BCR = “No” and	BCR = “No” and
	PFS.time >5yr	BCR.time >5yr	BCR.time >10yr

### A generic framework to infer reference-based and reference-free signatures

**Fig. 1:**
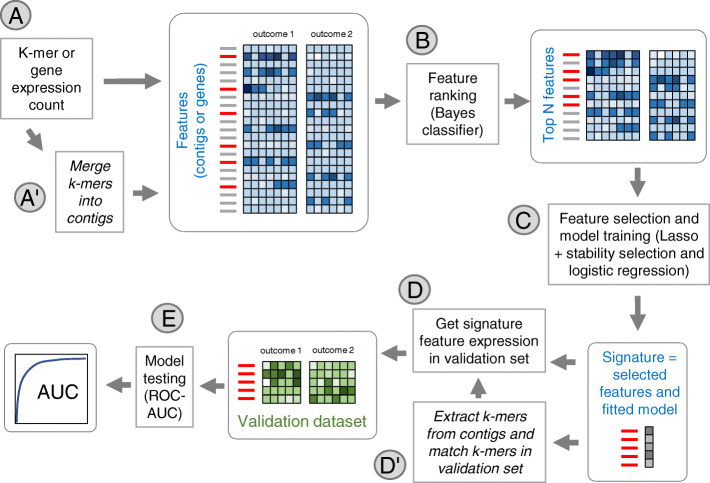
Uniform procedure for signature inference based on k-mer or gene expression. **a** The discovery matrix is built from normalized k-mer counts or gene expression counts. Samples are labelled by their outcome (risk or relapse) status. Normalization is performed as count per billion for k-mers or count per million for genes. **b** Features are ranked according to their F1-score computed by cross validation using a Bayes classifier (BC). The top 500 features are retained. **c** Among the top 500, features are selected using lasso logistic regression combined with stability selection. A logistic regression is tuned on the selected features. **d** Features from the signature are measured in the count matrix from an independent dataset. **e** Performance of the signature (selected features + tuned logistic regression) is evaluated using Area Under ROC Curve (AUC) on the validation dataset. To deal with the specificity of k-mer matrices, extra steps A’ and D’ are introduced: **a**’ the k-mer matrix in converted into a much smaller contig matrix by merging overlapping k-mers with compatible counts. **d**’ k-mers are extracted from the signature contigs and their counts in the validation matrix are aggregated

Risk and relapse predictors were derived using a combination of feature selection and supervised learning (Fig. 1). The predictive model was tuned over a discovery (or training) dataset and its performance was then evaluated on an independent validation (or testing) dataset, to avoid selection bias [30]. The same procedure was used for reference-based and reference-free models, however two extra steps were included to obtain and validate reference-free signatures. First a procedure was implemented to reduce the k-mer matrix using a sequence assembly-like algorithm to merge k-mers into contigs based on their sequence overlap and on the similarity of their count vectors. This step led to a contig count table an order of magnitude smaller than the initial k-mer count table (see “Results” section below). Feature selection and model fitting were performed over this contig table. A second adaptation was necessary to validate the reference-free signature in an independent dataset. This required extracting k-mers from both the signature and the sequence files of the independent set, and compute the signature expression in the independent set based on counts of matching k-mers. The pipeline is detailed in Methods. Note that we select features and train a predictive model only on the discovery dataset. The model is then applied to the validation set with no retraining (i.e. with the same coefficients) for an unbiased evaluation of the signature.

### Gene and k-mer count matrices

DEkupl-run [19] was used to produce gene and k-mer count matrices for each dataset. DEkupl-run converts FASTQ files to k-mer counts using Jellyfish [31], joins individual sample counts into a single count table and filters out low count k-mers. K-mer size was set to 31, lib _type to unstranded, and parameters min_recurrence and min_recurrence_abundance were set for each dataset as in Additional file 4: Table S1. K-mer size was set to 31 as commonly adopted for human transcriptome applications [19, 32]. Note that contrary to TCGA-PRAD, ICGC-PRAD uses stranded RNA-seq libraries. However we could not use this information as signatures were produced from unstranded libraries. We thus built all k-mer tables in canonical mode, which amounts to consider all libraries as unstranded. Gene expression was computed using Kallisto v0.43.0 [32] with Gencode V24 as a reference transcriptome. Gene-level counts were obtained by summing counts for all transcripts of each gene. Gene expression matrices were submitted to the same recurrence filters as k-mer tables to remove low expression genes. After count tables were generated and filtered, the k-mer merging and differential expression analysis module of DEkupl-run were not used. Instead, tables were further processed as explained below.

### Reduction of k-mer matrix via contig extension

**Fig. 2:**
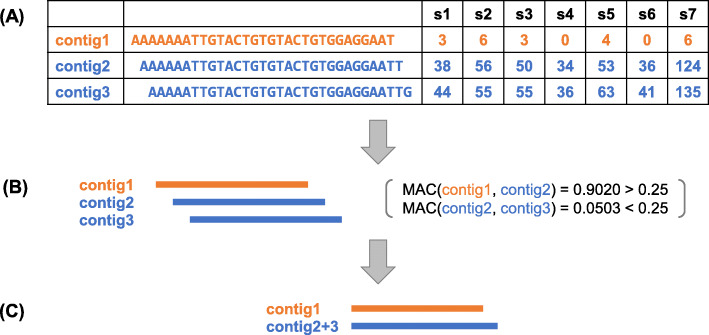
Merging procedure of 3 example contigs: **a** Count table of contigs in samples. Both pairs *(contig1, contig2)* and *(contig2, contig3)* have good overlaps shifting by only one nucleotide, but the sample count vectors of *contig1* and *contig2* are not compatible. **b** Merging intervention considering sample count compatibility between contigs. The mean absolute contrast (MAC) is calculated for each pair, and merging of *(contig1, contig2)* is rejected due to a MAC value exceeding threshold. **c** The resulting contigs are the initial *contig1* and the merged contig from the initial *(contig2, contig3)* pair

k-mer occurence tables were converted into contig occurence tables using an extension procedure similar to that described in Audoux et al. [19]. We define here as contig any sequence produced by merging 1 or more k-mers. Briefly, contigs overlapping by (k-1) to (k-15) nucleotide were iteratively merged into longer contigs till any of the following condition was encountered. In a straightforward case, extension stops when no more overlapping contig is available. Alternatively, extension stops when ambiguity is introduced i.e. when competing extension paths occur. Lastly, we applied here an intervention not included in Audoux et al. [19] by considering sample count compatibility between contigs, as shown in Fig. 2. Sample count compatibility is measured by the mean value of absolute contrast (MAC) between the counts of the two contigs across all samples, i.e.

$$\text{MAC}\left(\mathbf{c}_{\mathbf{1}},\mathbf{c}_{\mathbf{2}}\right)=mean_{s \in \{samples\}}\left(\left|\frac{c_{1,s}-c_{2,s}}{c_{1,s}+c_{2,s}}\right|\right) $$

where **c**_**1**_ and **c**_**2**_ are count vectors of two contigs to be merged, and *c*_1,*s*_ and *c*_2,*s*_ are counts in sample *s* from the corresponding count vectors. The extension is rejected if MAC>0.25. In this way, all contigs are guaranteed to have member k-mers with consistent sample count vectors. After the merging procedure, the new contig’s sample count vector is set to the mean of composite k-mer’s sample count vectors.

### Count normalization

To account for differences in sequencing depth among samples, we applied a normalization step on feature counts (genes or contigs) in discovery and validation datasets. Each feature count in a sample is divided by the sum of all feature counts in this sample, then multiplied by a constant base number:

$$e_{f,s} \leftarrow \frac{e_{f,s}}{{\sum\nolimits}_{f\in\{features\}}e_{f,s}} \cdot C_{b}, $$

where *e*_*f*,*s*_ refers to count of feature *f* in sample *s*, and *C*_*b*_ is the base constant. For genes, *C*_*b*_=10^6^ resulting in a conventional count per million (CPM) normalization, while for contigs, we used *C*_*b*_=10^9^, or count per billion (CPB). For contigs, normalization is applied on the contig count table produced after contig extension and for genes it is applied on the recurrence filtered gene expression matrix.

### Univariate features ranking

Given the limited number of samples, it was necessary to reduce the number of features (genes or contigs) in the dataset. We discarded irrelevant features to focus on a subset of 500 top candidates for subsequent feature selection. To rank features, we selected a Bayes classifier because the C++ implementation of this classifier was the fastest to run among several available feature ranking tools. We did not try to optimize this part to avoid biasing the comparison towards gene-based or gene-free methods. In detail, we performed prediction of status (risk/relapse) using a Bayes classifier on each independent feature, after log transformation of the normalized counts (after adding an offset 1 to avoid numerical problem). To assess the quality of the prediction, we computed the average *f*_1_ score by 5-fold cross validation ($f_{1} = \frac {2 \cdot \text {precision} \cdot \text {recall}}{\text {precision} + \text {recall}} $, where precision=*TP*/(*TP*+*FP*) and recall=*TP*/(*TP*+*FN*) and *FP*,*TP*,*FN* are respectively the False Positive, True Positive and False Negative). In cases where 5-fold cross-validation returned an undefined value, *f*_1_ score was set to 0 (the worst). The average *f*_1_ score was used to rank features. The Bayes classifier implementation was taken from the MLPack library [33].

### Feature selection, model fitting and predictor evaluation

To select a subset of non-correlated features (genes or contigs) among the top 500 candidates, we performed penalized logistic regression using the implementation from the glmnet R package [34]. We implemented stability selection [35]: only features selected with a frequency of being selected above 0.5 upon 2000 resamples of the input dataset were retained. To evaluate the performance of the selected features on the discovery (training dataset), we fitted a logistic regression and computed the area under the ROC curve (AUC) using a 10-fold cross validation scheme, repeated 20 times, as implemented in the caret package [36]. To handle imbalanced datasets, we included optional oversampling and downsampling in our evaluation procedures [37]. We also computed the Precision-Recall AUC, a more informative metric than the ROC AUC when evaluating binary classifiers on imbalanced datasets [38]. To assess the performance of the signature on the external validation datasets, we fitted a logistic regression on the whole discovery dataset and applied the predictor to the validation datasets. In the reference-free approach, some features present in the signature were not found in the validation (see below). In this case, the coefficient of the logistic regression corresponding to missing features were set to zero. Signature contigs were annotated through BLAST alignment *vs*. Gencode V34 transcripts. HGNC symbols for signature genes were obtained from the Ensembl EnsDb.Hsapiens.v79 R package [39].

### Matching signature contigs in the validation cohort

**Fig. 3:**
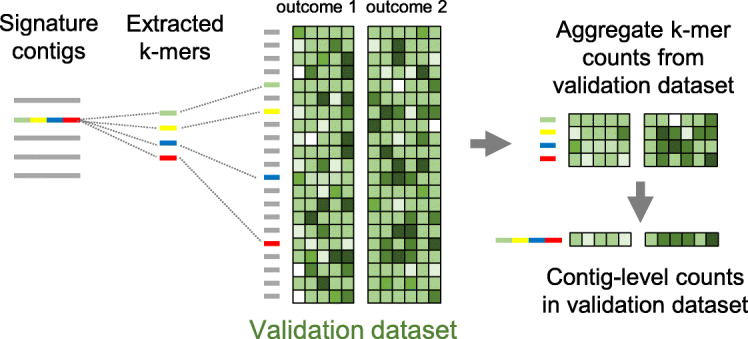
Procedure for inferring signature contig expression in an independent validation dataset. The colored contig from the signature is quantified in the validation cohort by extracting all its constituent k-mers and retrieving the corresponding k-mer counts from validation k-mer count matrix. The count vector of the contig in each sample of the validation dataset is taken as the median of counts for k-mers in this sample

To measure contig expression in the validation cohort we implemented the procedure schematized in Fig. 3. The procedure comprises two main steps: (1) all k-mers from signature contigs were extracted and identified in the k-mer count matrix generated from the validation cohort and (2) the resulting sub-matrix was used to estimate each contig’s expression in the validation cohort, measured for each sample as the median of extracted k-mer counts.

## Results

### A reference-free risk signature for prostate cancer

**Fig. 4:**
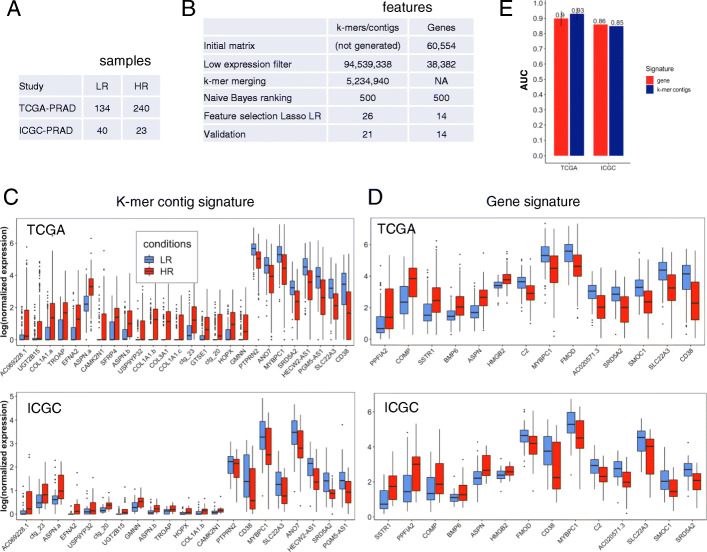
Risk signatures generation and analysis. **a** Characteristics of prostate tumor RNA-seq datasets. **b** Result of filtering procedure on the k-mer and gene matrices for risk analysis. Expression of risk signature elements in LR and HR samples in the TCGA-PRAD and ICGC-PRAD cohorts **c** k-mer contig signature; **d** Gene signature. **e** Signature performances for risk prediction in the TCGA-PRAD and ICGC-PRAD cohorts

We first applied the gene-free and gene-based signature discovery procedures detailed above to infer PCa risk signatures. The k-mer table for 374 TCGA-PRAD risk-labelled samples (Fig. 4a) had 94M k-mers after low count filtering. The merging step reduced it to 5.2M contigs, i.e. achieving a considerable 18-fold reduction in size (Fig. 4b). Contig sizes (mean=49nt, median=34nt, Table 3) were small relatively to a typical human RNA, which is characteristic of the adopted contig extension procedure [19] (see “Reduction of k-mer matrix via contig extension” section).

**Table 3:** Contig sizes (Risk model)

	After k-mer merging	After Bayes classifier ranking
Mean contig size (nt)	49.1	189
Median contig size (nt)	34	61

The 5.2M contig matrix and the 38k gene expression matrix were submitted to screening using univariate Bayes classification and the top scoring 500 features were retained for feature selection and model fitting. Interestingly, the 500 top scoring contigs were significantly longer than prior to selection (median 61nt vs. 34nt, Table 3), suggesting the procedure tended to eliminate spurious short contigs.

Finally, Lasso logistic regression produced a reference-free signature of 26 contigs and a reference-based signature of 14 genes (Fig. 4b). Ten-fold cross validation performances of both signatures were very high on the discovery dataset (0.90 and 0.93 for genes and k-mers, respectively) (Fig. 4e), which is an over-estimated performance since features here were tested on the same dataset used to select features [30]. PR-AUC and ROC-AUC on different sampling techniques to adjust the class distribution of a dataset are also presented in Additional file 4: Table S2. These results lead to the same conclusion as the ones presented in (Fig. 4e).

Figure 4c shows the 26 contigs in the reference-free risk signature and their abundance distribution in LR and HR samples. 24/26 contigs mapped Gencode transcripts from 21 unique genes (Additional file 1). Eleven of the 21 genes were also found in a list 180 genes compiled from published PCa outcome signatures (Additional file 2), which is a highly significant enrichment (*P*-value = 7.9e-9, Fisher’s exact test), especially when considering that no gene information was used to infer our signature. The gene and contig signatures involved five shared genes: MYBPC1, ASPN, SLC22A3, SRD5A2 and CD38 (Additional file 2, Fig. 4c and d). The first four genes are part of published prostate risk signatures. CD38 is particular in that it is the most downregulated in both signatures and it is not part of previous signatures. However, downregulation of this gene has been associated with poor outcome in prostate cancer [40], supporting its status as a high risk biomarker. Risk signature contigs mapped at least five other genes with established driver roles in PCa or other cancers: CAMK2N1 [41], COL1A1 [42], GTSE1 [43] and PTPRN2 [44], supporting the relevance of these sequence contigs in PCa etiology.

Of the two contigs that did not map any Gencode transcript, one aligned to an intron of GMNN (ctg_20), a gene also mapped by an exonic contig, the other an intron of LDLRAD4 (ctg_23). Contig ctg_23 corresponds to a 1.29 kb spliced transcript located between exons 4 and 5 of LDLRAD4 and is strongly upregulated in HR samples, as displayed in the Integrative Genomics Viewer (IGV) [45] in Additional file 4: Figure S1. Although ctg_23 partly maps short annotated LDLRAD4 isoforms, its expression seems unrelated to that of the longer LDLRAD4 transcripts whose coverage in flanking exons is 4-6 times lower than ctg_23 (Additional file 4: Figure S2.) Therefore ctg_23 likely comes from an independent lncRNA. The host gene LDLRAD4 is a negative regulator of TGF-beta signaling with roles in proliferation and apoptosis and was recently associated to negative outcome in other tumor types [46, 47]. Lastly, one contig (ctg_11, EFNA2) was probably misassigned to the EFNA2 gene since it maps to a highly expressed discrete area just 3’ of EFNA2 while EFNA2 seems silent. Thus ctg_11 probably comes from an independent lncRNA as well (Additional file 4: Figure S3).

To assess the replicability of risk signatures, we evaluated their performance in the ICGC-PRAD independent dataset. To this aim, we developed a specific procedure to estimate the expression of an arbitrary sequence contig across datasets using matched k-mers (see “Materials and methods” section). The 26 contigs represented 1444 k-mers, of which 97% were present in the ICGC-PRAD validation dataset. Overall 5 contigs (SFRP4, GTSE1, COL3A1, COL1A1.a, COL1A1.c) could not be quantified in the validation set due to lack of supporting k-mers (see Fig. 4b and c). In spite of this, the reference-free signature had similar performance in the validation set as the reference-based signature (0.85 and 0.86 respectively, Fig. 4e), although the later did not sustain any loss when transferred to the independent cohort (Fig. 4b). High prediction AUCs observed in the independent validation cohorts indicate a strong replicability of both the reference-free and reference-based risk signatures.

### Relapse signatures contain key PCa drivers

**Fig. 5:**
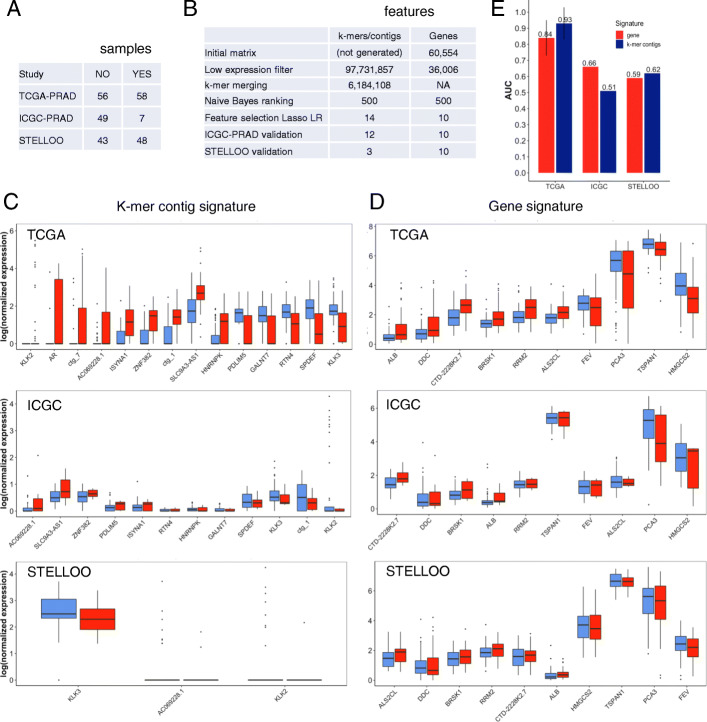
Relapse signatures generation and analysis. **a** Characteristics of prostate tumor RNA-seq datasets. **b** Result of filtering procedure on the k-mer and gene matrices for relapse analysis. Expression of relapse signature elements in LR and HR samples in the TCGA-PRAD, ICGC-PRAD and STELLOO cohorts **c** k-mer contig signature; **d** Gene signature. **e** Signature performances for relapse prediction in the TCGA-PRAD, ICGC-PRAD and STELLOO cohorts

For relapse prediction, we distinguished patients with biochemical relapse within less than 2 years and patients with no BCR after 5 years or longer. Application of the gene-free and gene-based signature discovery procedures to relapse prediction produced a 14-contig reference-free signature and a 10-gene reference-based signature (Additional file 2, Fig. 5b, c and d). The reference-free signature was populated by obvious PCa drivers. Strikingly, 3 contigs matched KLK2, AR and KLK3, which are among the most important genes in PCa onset and progression [48], the androgen receptor (AR) and two of its main targets, KLK2 and KLK3, the later encoding the PSA protein (Fig. 5c). Another contig matched SPDEF, a gene whose loss is associated to PCa metastasis [49].

Contigs matching KLK2 and AR were overexpressed 23-fold and 7-fold, respectively in relapsed patients while the contig matching KLK3 was depleted 1.8 fold. The AR contig matches exon 1 of AR and contains an non-templated poly-A end but no visible polyadenylation signal. The KLK2 contig is intronic and harbours a common SNP (rs62113074). The KLK3 contig is located in a distal part of the 3’ UTR region present only in longer isoforms of KLK3. Its lower expression in relapsed patients was unexpected as low expression of PSA is usually associated to a lower risk. It is possible though that only this longer isoform is depleted in relapsing samples. The expression boxplot shows the KLK2 contig occurs only in a few outlier patients while the AR and KLK3 contigs are common (Fig. 5c). The contig matching SPDEF is a special variant of the 3’ exon including two nonsynonymous SNPs. The SPDEF gene as a whole was highly expressed in both relapse and non-relapse samples but the contig expression was twice lower in average in relapse samples. Two contigs matched no known transcript: ctg_7 is a low complexity sequence of unknown origin and ctg_1 matches an intron of RPL9.

The contig matching lncRNA AC069228.1 also raised our attention since AC069228.1 is the only gene mapped by contigs in both relapse and risk signatures. The AC069228.1 lncRNA is antisense of PPFIA2, a protein tyrosine phosphatase that is itself an alleged urine biomarker of PCa [50]. The contigs from risk and relapse models match different regions of AC069228.1 (Figure S4). One is spliced, the other is a continuous 864 bp segment of a long exon. In both cases, a negative outcome (HR or relapse) is associated to a clearly higher expression of the contig, while the antisense gene PPFIA2 does not appear to follow the same trend (Figure S4).

Of note, the 10 genes in the reference-based signature were also clearly PCa-related: one was the major PCa biomarker PCA3 [51] and 5 others (DDC, RRM2, FEV, TSPAN1, HMGCS2) are involved in PCa etiology [52–56]. Therefore both gene-based and gene-free relapse signatures were significant in terms of PCa related functions of their component genes or contigs.

### Relapse signatures do not accurately classify independent cohorts

Contrary to the risk signatures, relapse signatures showed little overlap with each other and with published PCa signatures (Additional file 2). Only PCA3 and KLK2 were found in prior signatures [16, 57] and the only gene found shared between relapse and risk signatures in this study was AC069228.1. The poor overlap in this study was not unexpected as the discovery samples for risk and relapse information were quite disjointed and not always consistent: for instance only 25% of the high risk samples were labelled for relapse and 28% of these did not relapse. Conversely, 51% of non-relapse patients were labelled as HR. Therefore risk and relapse classifiers were trained to recognize quite different phenotypes.

As in the risk model, both reference-based and reference-free signatures had excellent cross-validation performance on the discovery set (AUC of 0.84 and 0.93 respectively, Fig. 5e). However this should again be considered as an overly optimistic estimation due to the experimental design. Indeed, performances of both relapse signatures on the ICGC-PRAD and Stelloo validation sets were much lower (AUC 0.51 to 0.66), bordering randomness and confirming overfitting of the trained signatures. Substituting the logistic Regression classifier by Random Forest, or Boosted Logistic Regression did not improve performance of either model (Table S3). The reference-based model performed slightly better over ICGC-PRAD, and the reference-free model was slightly better over the Stelloo dataset (Fig. 5e). Furthermore, several genes and contigs in the discovery signatures had inconsistent expression variations in the validation datasets (Fig. 5c and d, Additional file 3). Overall two genes from the reference-based signature (ALB and CTD-2228K2.7) and 5 contigs from the reference-free signature (KLK2, AC069228.1, PDLIM5, RTN4, ctg _1) changed logFC sign between the discovery and either validation cohort. This problem, which was not observed in risk models, underlines the poor replicability of the relapse signatures, whether or not reference-free.

Low replicability of the relapse model may be caused in part by weaknesses in validation datasets: the ICGC dataset had only 7 samples labelled for relapse (Fig. 5a) and the Stello dataset had very low coverage (Fig. 5a) which caused considerable loss when computing contig expression. Only three of the 14 signature contigs (AC069228.1, KLK2 and KLK3) could be quantified in the Stelloo dataset (Fig. 5b and c). Yet, we note that in spite of this loss the reference-free model still outperformed the reference-based model on this set (AUC of 0.62 vs. 0.59, Fig. 5e). Other limitations of the relapse model are addressed in the discussion.

## Discussion

### Properties of reference-free signatures

We evaluated here a method for building transcriptome classifiers that are totally reference-free, i.e. that do not require prior knowledge of genes or genome. The major interest of this approach lies in its ability to discover and incorporate in models previously unknown RNA biomarkers. Multiple examples exist of such disease-specific RNAs produced by genome alterations or deficient RNA processing and we hypothetized their inclusion in predictive models would be beneficial [18]. Applying a reference-free strategy to PCa outcome prediction, we obtained signatures made of short RNA contigs (median size 33 to 45 nt). These contigs are not full transcript models as can be produced by usual de novo assembly procedures. Instead, they often match SNPs or splice variants thus describing specific genetic or transcriptional events enriched in a patient group. Our strategy thus identifies RNA variations independently instead of lumping them into a full transcript model. Yet, the mapped genes were highly relevant to PCa etiology and included known cancer drivers LDLRAD4, GMNN, COL1A1, CD38, PTPRN2, GTSE1 and CAMK2N1 in the risk signature and KLK2, AR, KLK3, SPDEF in the relapse signature. Furthermore the risk signature comprised contigs matching two potential novel lncRNAs, located within LDLRAD4 and immediately downstream of EFNA2.

To our knowledge the only other software using a reference-free approach for inferring predictive signatures is Gecko [21]. Gecko uses machine learning (genetic algorithm) directly on the k-mer count matrix while we first reduce the matrix by grouping k-mers into contigs, before classification and machine learning. This enabled us to produce a signature composed of sequences larger than k, hence easier to interpret and quantify in an independent dataset.

Transferring a reference-free model to a new dataset is challenging. This requires that important features, such as SNPs, are precisely evaluated in the independent dataset. To this aim, we transferred signatures between datasets based on exact k-mer matches. As k-mer contents vary a lot between library preparation protocols, we expected this strategy to show poor sensitivity when discovery and validation datasets differed substantially. Indeed, transfer of signatures trained on the TCGA-PRAD dataset to the low coverage Stelloo dataset caused the loss of a majority of contigs. However, in this particular case, the remaining contigs were sufficient to maintain a prediction performance at the same level as that of the gene-based signature.

### Performances and generalization issues

To compare the reference-free and reference-based strategies, a common evaluation framework was adopted. For both risk and relapse predictions, performances of the reference-free classifiers were on a par with that of reference-based classifiers. However while risk signatures showed satisfying reproducibility, relapse signatures performed poorly in independent datasets.

A possible reason for the low performance of relapse models is our grouping of patients in discrete relapse and non relapse categories as done in other studies [9, 13, 15, 16]. This allowed us to address relapse prediction using the same logistic regression method as for risk, however this meant valuable patient information was left unused. A more accurate prediction of relapse may be achieved using survival models [10, 12, 14, 57, 58]. Adaptation of survival analysis tools to large k-mer matrices require additional developments that are certainly worth considering in the future.

A more general concern with relapse analysis is related to difficulty of predicting an outcome occurring several years after a sample is biopsied and analyzed. There might just be too little information available in the training data to infer a reliable classifier, a problem that is independent of the use of contigs or genes. However, both gene-level and contig-level signatures were highly enriched in PCa driver genes, which suggests information about tumor progression was indeed present in the primary tumor biopsy. The key problem with relapse analysis was more likely related to sample heterogeneity. The diversity of relapse mechanisms was not properly represented in a training set of 100 patients as we used here. Patient stratification have been proposed to deal with sample heterogeneity in omics data [59, 60]. Adaptations of these solutions to large k-mers matrices will also be considered in the future.

## Conclusion

For prediction of PCa risk and relapse, reference-free classifiers did not significantly outperform reference-based classifiers, however they incorporated a distinct set of RNA sequences including unannotated RNAs and novel variants of annotated RNAs. It is likely that with other diseases and datasets, novel biomarkers will be identified with an even greater impact on prediction performance. The reference-free approach will be of particular interest in problems where unknown RNAs are expected to play an important role, such as when studying rare diseases, poorly studied tissue types or when analysing dual human-pathogen RNA-seq samples. Our strategy also permits to infer efficient transcriptome classifiers in species lacking an accurate genome or transcriptome reference.

## Supplementary Information

**Additional file 1** Contig sequences and mapping locations in the risk and relapse signatures.

**Additional file 2** Published PCa risk and relapse signatures. Genes in common between published and this publication’s signatures.

**Additional file 3** Contents and expression characteristics of all signatures in the discovery and validation datasets.

**Additional file 4** Supplementary figures and tables.

## Data Availability

The codes to reproduce the experiments are available on GitHub at: https://github.com/i2bc/PCa-gene-based_vs_gene-free.

## Abbreviations

AUC: Are under the ROC curve
BCR: Biochemical relapse
HR: High risk
lncRNA: Long non-coding RNA
LR: Low risk
MAC: Mean absolute contrast
PCa: Prostate cancer
PSA: Prostate-specific antigen
RNA-seq: RNA sequencing
TNM: Tumour node metastasis
