# Supplementary material for: Reference-free transcriptome signatures for prostate cancer prognosis
Source: BMC Cancer. 2021 Apr 12;21:394. doi: 10.1186/s12885-021-08021-1 (PMC8040209; doi:10.1186/s12885-021-08021-1)
Supplement: Supplementary file 4 — Additional file 4 Supplementary figures and tables. [file 12885_2021_8021_MOESM4_ESM.pdf]

Reference-Free Transcriptome Signatures for Prostate Cancer Prognosis

Ha T.N. Nguyen[1], Haoliang Xue[1], Virginie Firlej[2], Yann Ponty[3], Melina Gallopin[1] and Daniel Gautheret[1]

[1] Institute for Integrative Biology of the Cell, UMR 9198, CEA, CNRS, Université Paris-Saclay, Gif-Sur-Yvette, France.

[2] Institute of Biology, Université Paris Est, Créteil, France.

[3] LIX, UMR 7161, Ecole Polytechnique, Institut Polytechnique de Paris, Palaiseau, France.

Supplementary Figures and Tables

Table S1. Filtering parameters for count tables

	Analysis	min_recurrence	min_recurrence_abundance
TCGA-PRAD	Risk	3	10
ICGC-PRAD		5	5
TCGA-PRAD	Relapse	3	5
ICGC-PRAD		4	2
STELLOO		3	5

Table S2. PR-AUC and ROC-AUC metrics computed for risk and relapse signatures using oversampling and downsampling.

				gene	kmers
risk	TCGA (10CV)	no correction	ROC AUC	0.9 +/-0.05	0.93+/-0.04
			PR AUC	0.91+/-0.02	0.92+/-0.02
		downsampling	ROC AUC	0.9+/-0.05	0.92+/-0.04
			PR AUC	0.91+/-0.03	0.92+/-0.03
		upsampling	ROC AUC	0.90+/-0.05	0.93+/-0.04
			PR AUC	0.91+/-0.03	0.92+/-0.02
	ICGC	no correction	ROC AUC	0.86	0.85
			PR AUC	0.93	0.92
		downsampling	ROC AUC	0.84	0.85
			PR AUC	0.92	0.91
		upsampling	ROC AUC	0.86	0.84
			PR AUC	0.93	0.91
relapse	TCGA (10CV)	no correction	ROC AUC	0.82+/-0.12	0.93+/-0.1
			PR AUC	0.65+/-0.12	0.63+/-0.28
		downsampling	ROC AUC	0.81+/-0.12	0.93+/-0.09
			PR AUC	0.65+/-0.12	0.58+/-0.3
		upsampling	ROC AUC	0.81+/-0.13	0.93+/-0.09
			PR AUC	0.65+/-0.12	0.65+/-0.3
	ICGC	no correction	ROC AUC	0.66	0.51
			PR AUC	0.1	0.11
		downsampling	ROC AUC	0.67	0.52
			PR AUC	0.1	0.11
		upsampling	ROC AUC	0.67	0.52
			PR AUC	0.1	0.11
	Stelloo	no correction	ROC AUC	0.6	0.62
			PR AUC	0.44	0.57
		downsampling	ROC AUC	0.6	0.62
			PR AUC	0.45	0.57
		upsampling	ROC AUC	0.6	0.62
			PR AUC	0.44	0.57

Table S3: PR-AUC and ROC-AUC metrics computed for relapse signatures using three different prediction models (Logistic regression, boosted logistic regression and random forests) combined to oversampling and downsampling.

			Gene			K-mer		
			Logistic regression	Boosted Logistic Regression	Random Forest	Logistic regression	Boosted Logistic Regression	Random Forest
TCGA (10CV)	no correction	ROC AUC	0.82+/-0.12	0.77+/-0.14	0.84+/-0.12	0.93+/-0.1	0.94+/-0.67	0.93+/-0.1
		PR AUC	0.65+/-0.12	0.59+/-0.14	0.68+/-0.11	0.63+/-0.28	0.72+/-0.11	0.63+/-0.3
	downsampling	ROC AUC	0.81+/-0.12	0.77+/-0.14	0.84+/-0.12	0.93+/-0.09	0.94+/-.07	0.93+/-0.09
		PR AUC	0.65+/-0.12	0.6+/-0.15	0.68+/-0.1	0.58+/-0.3	0.71+/-0.11	0.58+/-0.3
	upsampling	ROC AUC	0.81+/-0.13	0.77+/-0.13	0.84+/-0.13	0.93+/-0.09	0.94+/-0.07	0.93+/-0.09
		PR AUC	0.65+/-0.12	0.6+/-0.14	0.68+/-0.11	0.65+/-0.3	0.71+/-0.11	0.63+/-0.3
ICGC	no correction	ROC AUC	0.66	0.76	0.67	0.51	0.58	0.51
		PR AUC	0.1	0.08	0.09	0.11	0.11	0.11
	downsampling	ROC AUC	0.67	0.71	0.68	0.52	0.58	0.52
		PR AUC	0.1	0.08	0.1	0.11	0.1	0.1
	upsampling	ROC AUC	0.67	0.75	0.67	0.52	0.62	0.51
		PR AUC	0.1	0.08	0.1	0.11	0.11	0.11
Stelloo	no correction	ROC AUC	0.6	0.57	0.57	0.62	0.58	0.62
		PR AUC	0.44	0.46	0.45	0.57	0.57	0.58
	downsampling	ROC AUC	0.6	0.56	0.57	0.62	0.5	0.62
		PR AUC	0.45	0.46	0.44	0.57	0.5	0.57
	upsampling	ROC AUC	0.6	0.56	0.57	0.62	0.62	0.62
		PR AUC	0.44	0.46	0.46	0.57	0.58	0.57

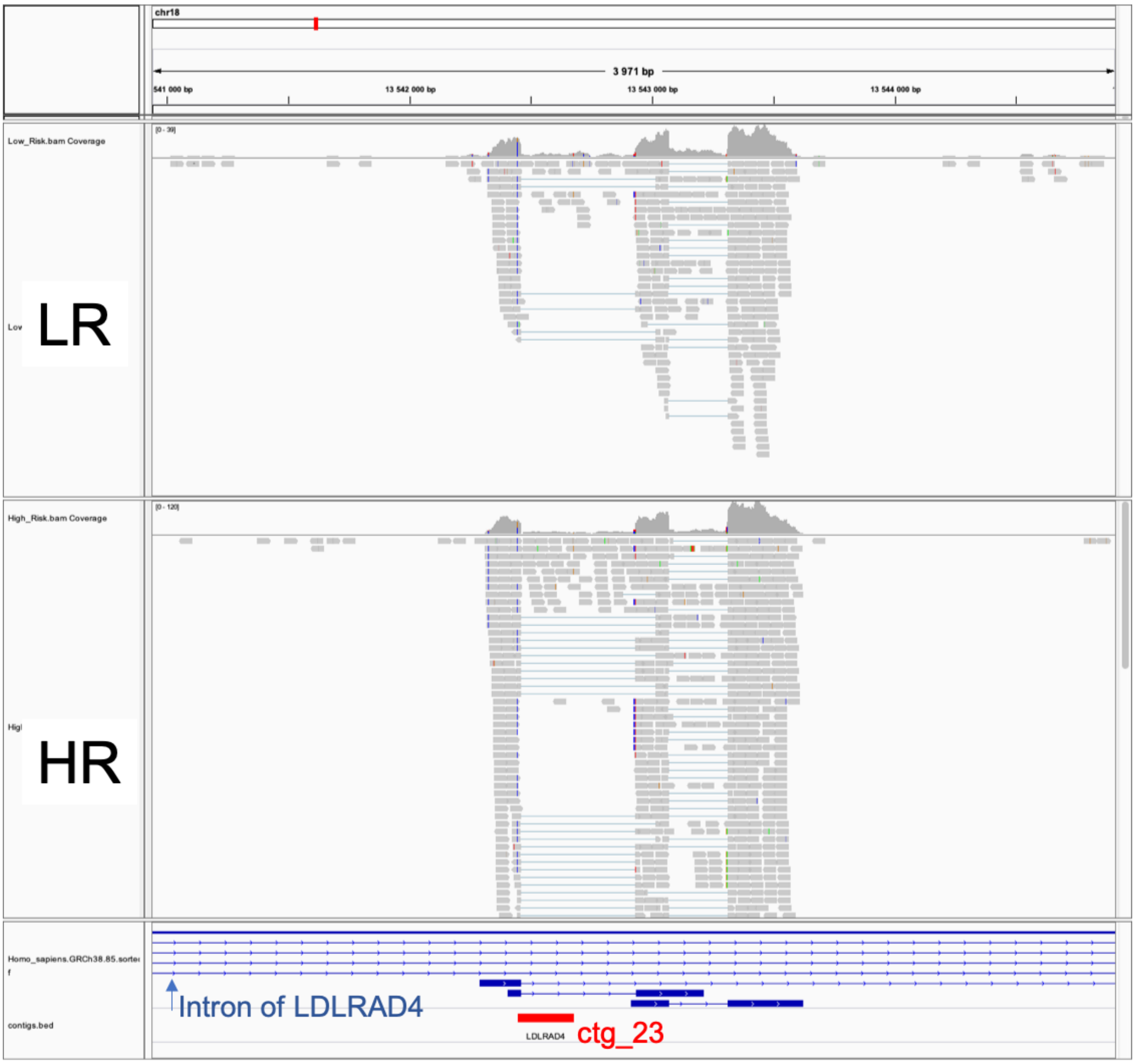

Figure S1. IGV view of RNA-seq reads from the TCGA-PRAD discovery set aligned at the genomic location of risk signature contig ctg_23 (red box). This contig is located in an intron of LDLRAD4. Frames LR and HR show reads sampled from all samples in the LR and HR subsets, respectively, at identical depth for each. Blue boxes and lines in the bottom frame correspond to Gencode annotations of LDLRAD4 transcript isoforms (thick lines: exons, thin lines with arrows: introns).

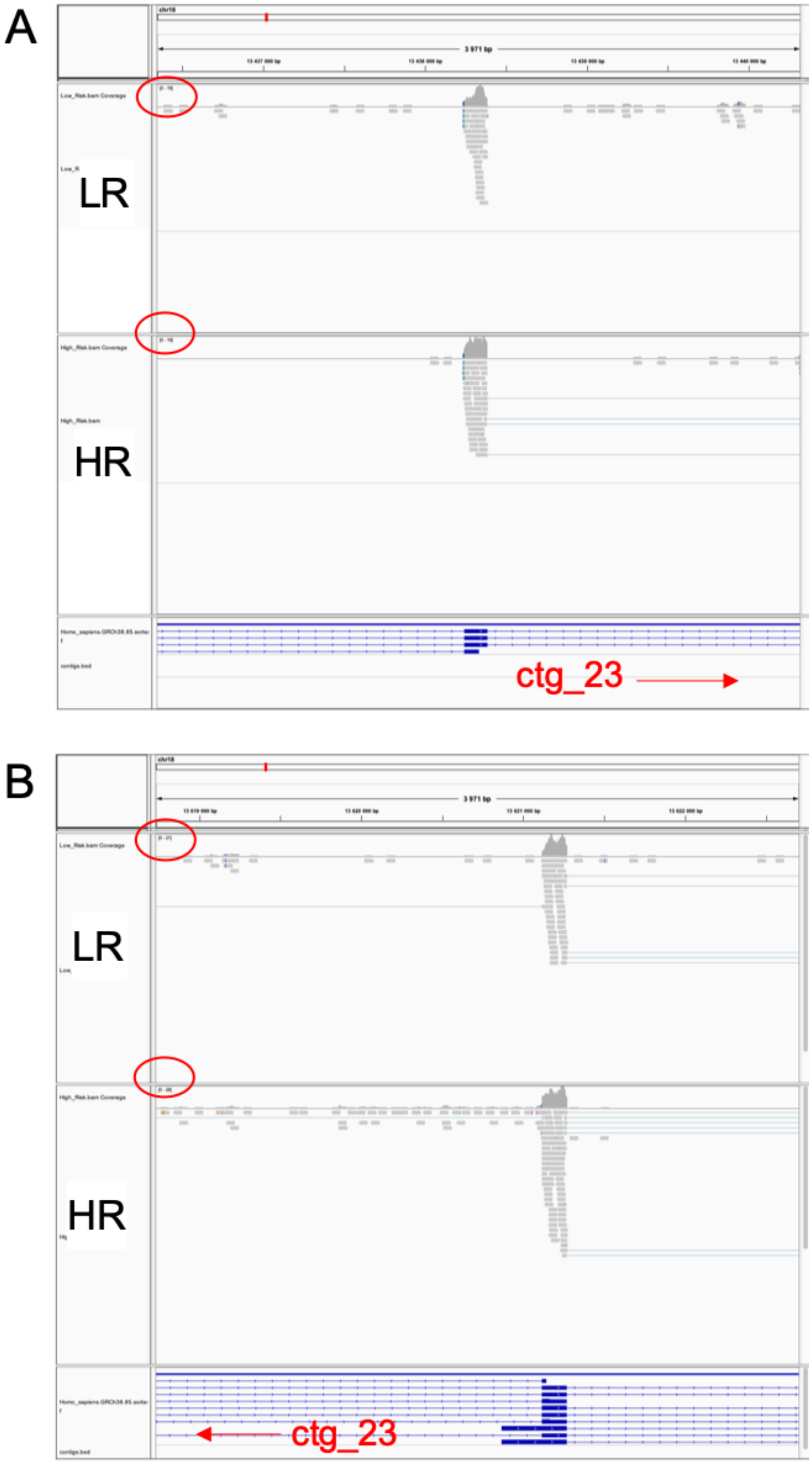

Figure S2. IGV view of RNA-seq reads aligned on the LDLRAD4 exons flanking signature contig ctg_23 on the left (A) and right (B) side of the genomic location of the contig. HR and LR frames are as described above. Note the coverage depth about 6 times lower than ctg_23 coverage in HR condition (red circles) and its lack of variation between LR and HR conditions.

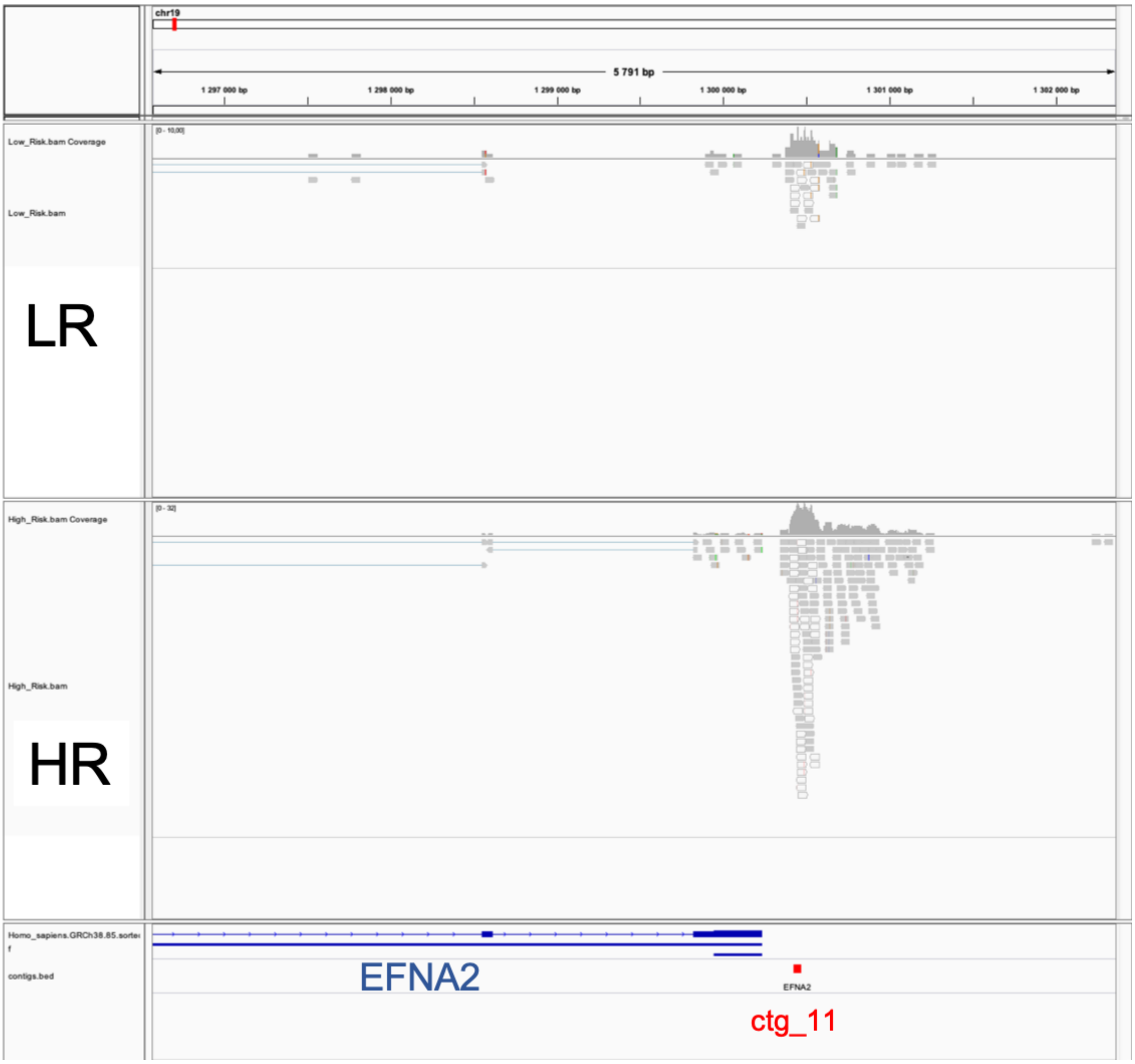

Figure S3. IGV view of RNA-seq reads aligned at risk signature contig ctg_11. Figure legend is as above. ctg_11 was assigned to EFNA2 based on an 3' extended isoform (not shown), but it appears it is more likely an independent transcript.

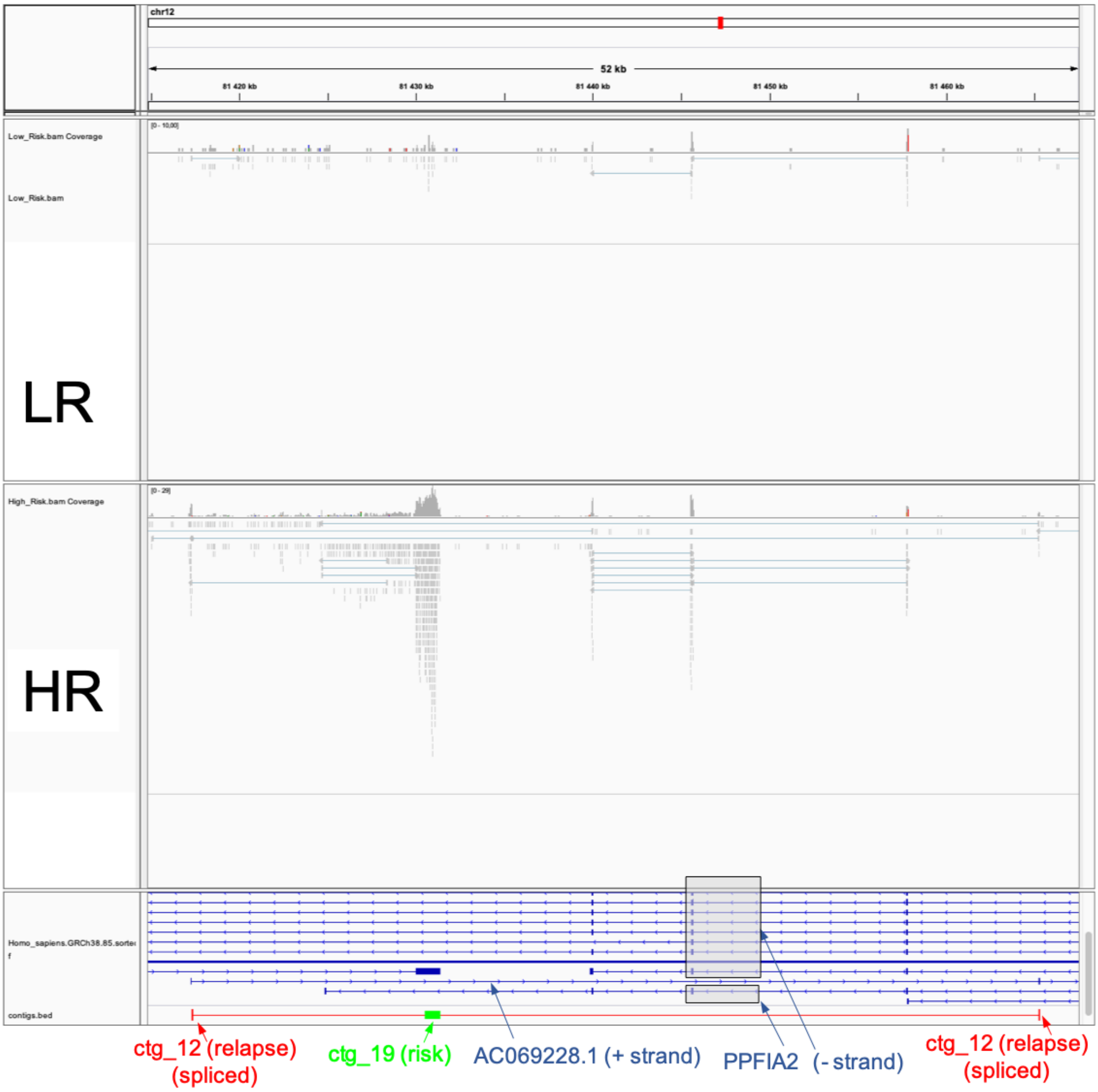

Figure. S4. IGV view of RNA-seq reads aligned at locus AC069228.1, where two signature contigs (ctg_19 from the risk model and ctg_12 from the relapse model) are aligned. Figure legend is as above. Contigs match two different transcripts of the AC069228.1 lncRNA gene, located antisense of gene PPFIA2 (boxed transcripts). In spite of the unstranded nature of aligned reads, mapping to AC069228.1 is unambiguous as only this gene has annotated exons at the corresponding locations.
